# Bevacizumab for Treatment-Refractory Pain Control in Neurofibromatosis Patients

**DOI:** 10.7759/cureus.933

**Published:** 2016-12-18

**Authors:** Xu W Linda, Lawrence D Recht

**Affiliations:** 1 Department of Neurosurgery, Stanford University School of Medicine; 2 Department of Neurology, Stanford University School of Medicine

**Keywords:** neurofibromatosis, bevacizumab, vegf, pain control

## Abstract

Objective: Chronic pain is a well-known morbidity associated with neurofibromatosis (NF) for which better therapies are needed. Surgery, radiation, and pain medications have been utilized, but often fail to relieve debilitating pain. One patient at our institution was noted to have near complete resolution of pain after treatment with bevacizumab for progressive neurologic deficit associated with NF2, suggesting its potential as an effective pain control method. We aim to better characterize the use of bevacizumab for pain control in this subset of patients.

Patients and Methods: We retrospectively reviewed 38 NF patients treated at our institution.

Results: Of the 38 total NF patients, we found that 63% reported chronic pain, with 18% reporting chronic opiate usage. Nine patients with chronic pain were considered for bevacizumab treatment and five went on to receive infusions. Of these patients, four out of five had previous surgical debulking and two out of five had previous radiation for attempted pain control. One patient had a lesion not amenable to surgery or radiation. Patients received a median of 13 cycles of bevacizumab, and four out of five patients reported a decrease in subjective pain. All patients that had pain relief had a relapse of pain symptoms when the dose was reduced or infusions were paused. Seventy-five percent were able to decrease opiate use. No major complications were noted. All five patients have elected to continue infusions for pain control.

Conclusion: Bevacizumab was, in general, well tolerated and should be considered as a treatment option in NF patients with chronic pain refractory or not amenable to surgical decompression and debulking, radiation, and pain medication.

## Introduction

Neurofibromatoses (NF) represent a family of neurocutaneous disorders characterized by the development of multiple neuronal tumors, including schwannomas and neurofibromas. Neurofibromas in these patients can be numerous and plexiform, involving multiple nerve roots at a time. In addition to neuropathy, these tumors can cause a significant pain burden for patients and represent a serious source of overall morbidity. All NF patients report more impairment from body pain compared to age-matched controls without NF [1-2]. In studies of pain in patients with NF1, 65% complained of pain related to peripheral nerve tumors [3] and 61% had chronic headaches [4].

The traditional method of pain control in the NF patient is to identify the most symptomatic lesion and attempt surgical resection and decompression of that specific lesion. However, surgical resection remains fraught with difficulties as lesions are often vascular and can cause significant intraoperative blood loss. Furthermore, invasion into the nerve by tumor leads to either incomplete resection or new postoperative neurologic deficits. Even with complete resection, 20% of lesions will recur at the same location. Furthermore, there are some locations, such as plexiform neurofibromas of the face, that are essentially unresectable [5-6]. Radiation has not been used traditionally for pain control in NF, but it has been described in the literature with some efficacy in peripheral nerve tumors in pain reduction and limitation of growth [7-9]. 

Other methods of pain control have been reported, primarily as case reports. Individual patients have benefited, for example, from ketamine, occipital nerve stimulator, physical therapy, pirfenidone, and thalidomide [6, 10-12]. Bevacizumab (Avastin), a monoclonal antibody to vascular endothelial growth factor (VEGF-A), is utilized in oncologic treatment for a variety of tumors to limit vascular permeability, reducing both tumor growth and edema. With the reduction in edema, many patients also experience a reduction in symptoms, such as pain from mass effect. At our institution, one patient with NF2 was noted to have near complete resolution of pain after beginning bevacizumab infusions for debilitating pain and deficits related to tumors already previously treated with surgery and radiation. Her gratifying response, in terms of pain control, led us to try this approach on other patients.

## Materials and methods

### Index patient case

A 34-year-old woman first presented in 2005 with a diagnosis of NF2. She underwent resection of a Grade 2 cervical ependymoma two years prior with subsequent fractionated radiotherapy. Her primary complaint was pain in the neck and bilateral shoulders. At the time she was taking ibuprofen, carisoprodol, and occasional hydrocodone/ acetaminophen. She previously tried fentanyl patches, amitryptiline, baclofen, and gabapentin. The following year her MRI remained stable, but she increased her hydrocodone/acetaminophen usage and developed new right-hand numbness. She was referred for acupuncture and cyclobenzaprine, which she self-discontinued later due to sleepiness. She subsequently tried desipramine and duloxetine, which produced mood alterations and were also stopped. Her hydrocodone/ acetaminophen usage increased over time from one tab a day to multiple tabs a day. 

She was hospitalized in 2008 briefly following a fall with a worsening of bilateral upper extremity numbness and pain. She was given a dexamethasone taper at that time with some improvement in pain. She continued to have lower extremity discoordination off steroids and developed a right upper extremity claw hand. Given her instability, she was fitted for a walker and a wheelchair. She also developed a left thigh neurofibroma that was painful to the touch and was referred back to Neurosurgery for evaluation. Surgery was deferred for concern that it may further worsen the neurologic deficit in her leg. In November 2009, the patient was wheelchair-bound from debilitating pain and frequent falls. She increased her opiate from occasional hydrocodone/ acetaminophen, soma, and ibuprofen in 2005 to oxycodone/ acetaminophen every four to six hours with supplemental oxycodone for breakthrough pain. The pain was not controlled with carisoprodol, fentanyl patch, amitryptiline, baclofen, gabapentin, cyclobenzaprine, duloxetine, and desipramine. On November 24, 2009, the patient began bevacizumab, 7.5 mg/kg every two weeks for three cycles, for tumor growth and pain control. In her first follow-up in January, she reported a subjective decrease in pain and improvement in clinical status, such that the patient was beginning to drive again. By June 2010, the patient reported significant improvement in pain and had begun walking again without the wheelchair. In September 2010, she reported walking daily. Her bevacizumab infusions were spaced to every four weeks. She reported using hydrocodone/acetaminophen, 0-3 times a day, and was no longer using oxycodone for breakthrough pain.

### Retrospective review

Under the Stanford University Hospital and Clinics IRB protocol 32041, we retrospectively identified 38 patients diagnosed with NF evaluated in the Stanford Neuro-Oncology clinic. Patient demographics, including age, gender, NF type, the presence of chronic pain at the time of first evaluation, and previous treatments for pain, were recorded. For those treated with bevacizumab, pain medications, start and stop dates for bevacizumab, a number of cycles, the presence of subjective pain reduction, time to pain reduction, and the clinical course after infusions were stopped were also recorded.  

## Results

Patient demographics for all patients with NF are summarized in Table 1. Nineteen patients carried a diagnosis of NF1 and 17 patients were labeled as NF2. Two patients had clinical findings suggestive of NF, such as schwannomatosis, but had not been officially categorized by type. Age ranged from 25 - 60 years old with a median age of 37. Twenty-four patients (63%) identified chronic pain as a component of their disease. Pain complaints involved the extremities in 11 (29%), back pain in seven (18%), flank or chest pain in three (8%), headache in four (11%), and the facial pain in two (5%). Over half of all NF patients had or were currently receiving pain treatments, with over half on current pain medications (55%) with 18% overall on opioids, a quarter (26%) with previous surgical intervention, and 13% with previous radiation treatment.  

**Table 1:** Patient Demographics

Patient Demographics (N = 38)		
	Number	Percentage
NF1	19	50%
NF2	17	45%
Male sex	22	58%
Female sex	16	42%
Chronic pain	24	63%
Location of Pain		
* Extremity*	11	29%
* Back Pain*	7	18%
* Flank/Chest*	3	8%
* Headache*	4	11%
* Face*	2	5%
Previous Pain Treatments		
* Resection*	10	26%
* Medication*	21	55%
* Radiation*	5	13%
Bevacizumab offered	17	45%
Bevacizumab received	10	26%
Bevacizumab given for pain	5	13%

Ten NF patients received bevacizumab infusions. Bevacizumab was administered for all patients every two to four weeks at a dose of 7.5 mg/kg. For each infusion, a complete blood count, chemistry panel, and urinalysis were obtained and vital signs taken for blood pressure. For five patients, the primary reason for treatment was a concern for tumor progression; for five patients, treatment was administered for tumor-related pain. In the five patients receiving bevacizumab for non-pain indications, two patients did have pain complaints prior to starting infusion, with one of these patients able to stop anti-inflammatories for burning leg pain four weeks after starting the infusions. The other patient did not have improved pain with the infusions.  

Characteristics of the five patients receiving bevacizumab for pain control are outlined in Table 2. Of these five patients, two were diagnosed with NF1 and three with NF2. Primary foci of pain were facial (two), pelvic and extremity (two), and neck and shoulders (two). Four patients had received prior surgical debulking for pain control and the remaining patient was not a surgical candidate for a plexiform facial neurofibroma. Three patients had prior radiation therapy. At the time of starting bevacizumab, four of five patients were taking chronic pain medications with all four using narcotics and three using multiple neuroleptic pain medications. Patients received bevacizumab overall for three months to five years with numbers of cycles ranging from six to 51 cycles. Four of five patients reported significant pain relief within three to eight weeks of the first infusion. Of the four patients who were on narcotics, two were able to stop all scheduled narcotics and one was able to reduce the dose to prn. Two patients were able to wean off neuroleptic agents as well. All those whose pain responded to bevacizumab had a recurrence of pain when cycles were stopped or the interval between infusions was extended. One patient developed hypertension and was started on Lisinopril, which provided adequate control. One patient had abdominal pain while on bevacizumab and infusions were stopped and then restarted without recurrence of pain. No other complications were noted. Four of five patients, including the index patient, are currently continuing bevacizumab infusions.  

**Table 2:** Five Patients Treated with Bevacizumab for Pain Four patients had previously had surgery and one was felt to be unresectable. Three had previously had radiation. Four were on narcotics. Patients were treated for three months to five years with four reporting decrease in overall pain.

Patient Number	NF Type	Age (years)	Gender	Pain Location	Prior Pain Treatments	Pain Medication Pre-BEV	BEV Start Date (Month-Year)	Last BEV Infusion (Month-Year)	Number of Cycles	Pain Response	Time to Response (weeks)	Pain Relapse	Pain Medication Post-BEV	Complications
1	2	43	F	Leg, Neck, Shoulders	Surgical resection; Radiation	Oxycodone scheduled and PRN Carisoprodol	11/2009	3/2015	52	Yes	6	Increase in pain while BEV paused for surgery	Oxycodone PRN	Abdominal pain resolved and able to restart treatment with no recurrence
2	1	57	M	Shoulder	Surgical resection; Radiation	Baclofen, Trileptal, Nortriptyline, Hydromorphone ER and PRN Pregabalin	8/2012	3/2015	18	Yes	8	Increase in pain when missed one infusion dose	Baclofen dose reduced Trileptal, Pregabalin, Dilaudid PRN	None
3	2	25	M	Facial	Surgical resection; Radiation	None	3/2013	4/2015	46	Yes	3	Increase in pain when decreased to q3 week intervals	Gabapentin	Hypertension on lisinopril
4	2	49	F	Pelvic and leg	Surgical resection	Oxycodone, Nabumetone, Carbamazepine, Amitryptiline	3/2014	4/2015	13	Yes	3	Increase in pain when decreased to q3 week intervals	Topiramate, Amitryptiline	None
5	1	47	M	Facial	Not a surgical candidate	Methadone, Gabapentin, Amitryptiline	9/2014	12/2014	6	Stable	-	Increase in pain 4 months after stopping infusion	Methadone, Gabapentin, Amitryptiline	None

## Discussion

Neurofibromatosis is a common neurocutaneous disorder associated with both central and peripheral tumors. Pain can frequently become the primary disease-related morbidity requiring multiple surgical interventions, radiation treatments, and pain medications. In our study, we found that 63% of patients complained of chronic pain and over half were taking pain medications. Many had already received prior surgery and radiation to attempt to alleviate pain. These results correlate well with previous studies showing that pain is reported by a majority of NF patients [1-4].

While surgery and radiation can both offer local control if one responsible lesion can be determined, the frequent presence of multiple or plexiform lesions often creates major difficulties in pinpointing just which one is the etiology. Even if a particular cause of pain is identified, surgery carries a significant risk of nerve damage and heavy blood loss [5-6]. Our index case reflects this scenario as surgery had already been performed on one lesion with significant residual pain and a second symptomatic lesion was felt to be not amenable to surgery because of the risk for furthering neurologic compromise. 

For the NF pain patient, therefore, a systemic treatment that could address complex or multifocal sources of pain with a low side effect profile would be of great benefit. Bevacizumab is an anti-VEGF-A agent that has been frequently used in other settings for tumors and radiation necrosis to reduce permeability and edema, often with an alleviation of associated pain [13-14]. Bevacizumab’s role in NF to date largely relates to its hearing sparing effect in NF2 [15], and up to now, using this agent for pain control has only been reported twice [16-17]. This report confirms in a larger group that for patients who have already failed multiple other methods for pain control, bevacizumab can be an effective pain treatment. Four of our five patients who had significant pain at the initiation of treatment reported decreased pain, and three out of those four were able to significantly reduce the dosage of other pain medications. Furthermore, all patients who responded noted increased pain once infusions were paused or the dose was decreased and all responded to reinstitution of drug. Because this is a retrospective review, no numeric value was assigned to pain scores before and after treatment in our records, so quantitative assessment of change in pain was limited. However, objectively, all patients reviewed here elected to continue with infusions and most decreased their opiate usage. Bevacizumab was well tolerated, even when administered for long intervals, with no significant complications noted. Of note, bevacizumab can be associated with hypertension, renal compromise, GI bleed, and wound healing issues [23] and is not recommended for patients who consider pregnancy, have planned surgeries within the next month, or have known open wounds or hemorrhages. Given these risks, patient selection to minimize potential drug complications is key. Future prospective studies could focus on appropriate patient selection, quantitative analysis of pain medication reduction, and pain score reduction. 

VEGF has been shown to be upregulated in neurofibromas and schwannomas [18-19]. There are at least two plausible complementary reasons for why bevacizumab was effective in these patients. The first concerns an ameliorative effect on tumor edema. In the case series reporting the use of bevacizumab in the treatment of acoustic neuromas and meningiomas in NF2 [15, 20], there was an association between changes in apparent diffusion coefficient values in MRI and responses, suggesting that this agent was producing its effect via reduction in overall edema and vascular permeability [15]. 

Experimental evidence exists as well supporting the contention that that VEGF also plays a direct role in pain sensation. In murine models, VEGF is upregulated in mechanically injured nerves [21]. In such models, administration of a VEGF-A inhibitor reduces the development of allodynia and hyperalgesia in the limb with the associated nerve injury, an effect that was dose-dependent [21]. In another study, administration of a VEGF inhibitor reduced the expression of receptors known to be involved in pain sensation in the dorsal horn of injured rats [22]. Thus, VEGF may play a direct role in tumor growth and edema but may be particularly useful in tumor-related pain as it may have a secondary role in the development of neuropathic pain and pain transmission as well.

## Conclusions

Chronic pain is prevalent in the NF population and is a source of significant morbidity. While previously reported in the context of controlling acoustic neuroma growth, we report a series of NF patients with refractory pain that was successfully treated with bevacizumab. Bevacizumab was overall well tolerated and all patients have elected to pursue continued therapy for pain control. We believe that bevacizumab should be considered for as an option for NF patients with refractory pain. 
